# Alcohol Consumption and Autoimmune Diseases

**DOI:** 10.3390/ijms26020845

**Published:** 2025-01-20

**Authors:** Sergio Terracina, Brunella Caronti, Marco Lucarelli, Silvia Francati, Maria Grazia Piccioni, Luigi Tarani, Mauro Ceccanti, Micaela Caserta, Loredana Verdone, Sabrina Venditti, Marco Fiore, Giampiero Ferraguti

**Affiliations:** 1 Department of Experimental Medicine, Sapienza University of Rome, 00161 Rome, Italy; sergio.terracina@uniroma1.it (S.T.); marco.lucarelli@uniroma1.it (M.L.); silvia.francati@uniroma1.it (S.F.); 2 Department of Human Neurosciences, Sapienza University Hospital of Rome, 00185 Rome, Italy; 3 Pasteur Institute, Cenci Bolognetti Foundation, Sapienza University of Rome, 00161 Rome, Italy; 4 Department of Maternal Infantile and Urological Sciences, Sapienza University of Rome, 00161 Rome, Italy; mariagrazia.piccioni@uniroma1.it (M.G.P.); luigi.tarani@uniroma1.it (L.T.); 5 SITAC, Società Italiana per il Trattamento dell’Alcolismo e le sue Complicanze, 00185 Rome, Italy; mauro.ceccanti@uniroma1.it; 6 Institute of Molecular Biology and Pathology (IBPM-CNR), 00161 Rome, Italy; micaela.caserta@cnr.it (M.C.); loredana.verdone@cnr.it (L.V.); 7 Department of Biology and Biotechnologies “Charles Darwin”, Sapienza University of Rome, 00161 Rome, Italy; sabrina.venditti@uniroma1.it; 8 Institute of Biochemistry and Cell Biology (IBBC-CNR), c/o Department of Sensory Organs, Sapienza University of Rome, 00161 Rome, Italy

**Keywords:** alcohol, immune system, autoimmunity, metabolism, inflammation

## Abstract

Alcohol is the second-most misused substance after tobacco. It has been identified as a causal factor in more than 200 diseases and 5.3% of all deaths and is associated with significant behavioral, social, and economic difficulties. As alcohol consumption may modulate the immune system’s regulatory mechanisms to avoid attacking the body’s tissues, it has been proven to play a dichotomic role in autoimmune diseases (ADs) based on the quantity of consumption. In this review, we report updated evidence on the role of alcohol in ADs, with a focus on alcohol addiction and the human biological immune system and the relationship between them, with alcohol as a risk or protective factor. Then, in this narrative review, we report the main evidence on the most studied ADs where alcohol represents a key modulator, including autoimmune thyroiditis, multiple sclerosis, rheumatoid arthritis, systemic lupus erythematosus, diabetes, allergic rhinitis, and primary biliary cholangitis. Alcohol at low–moderate dosages seems mostly to have a protective role in these diseases, while at higher dosages, the collateral risks surpass possible benefits. The specific mechanisms by which low-to-moderate alcohol intake relieves AD symptoms are not yet fully understood; however, emerging studies suggest that alcohol may have a systemic immunomodulatory effect, potentially altering the balance of anti-inflammatory innate and adaptive immune cells, as well as cytokines (via the NF-κB or NLRP3 pathways). It might influence the composition of the gut microbiome (increasing amounts of beneficial gut microbes) and the production of their fatty acid metabolites, such as short-chain fatty acids (SCFAs) and polyunsaturated fatty acids (PUFAs), as well as elevated concentrations of acetate, high-density lipoprotein (HDL), and nitric oxide (NO). Unfortunately, a definite acceptable daily intake (ADI) of ethanol is complicated to establish because of the many mechanisms associated with alcohol consumption such that despite the interesting content of these findings, there is a limit to their applicability and risks should be weighed in cases of alcoholic drinking recommendations. The aim of future studies should be to modulate those beneficial pathways involved in the alcohol-protective role of ADs with various strategies to avoid the risks associated with alcohol intake.

## 1. Alcohol Addiction

After tobacco, alcohol is the most misused substance, and it has been identified as a causal factor in more than 200 diseases and 5.3% of all deaths (https://www.who.int. Accessed on 14 November 2024). Furthermore, alcohol addiction has been associated with significant behavioral, social, and economic difficulties. Despite the common knowledge about the dangers of alcohol usage, marketing and advertising associated with increased drinking intentions, consumption, and harmful drinking are still common [1,2]. Researchers agree that stronger interventions, particularly those targeted to younger individuals, are needed to reduce the significant global health loss attributable to alcohol [3,4].

A definite acceptable daily intake (ADI) of ethanol has not been established, and some studies controversially recommend a variety of dosages of ADI, whilst others regard any intake as dangerous [5]. Alcohol addiction increases the risk of hypercortisolism, melanoma and cancer of the oral cavity and pharynx, esophagus, colorectum, liver, larynx, female breast, pancreas, and prostate [6,7,8,9]. Recent studies found that alcohol use disorders (AUDs) have a complex interplay with nutrition: AUD is associated with an increased risk of malnutrition, while impairments in nutritional status can be detrimental to physical health and to the prospects of recovery and treatment outcomes [10,11]. Furthermore, chronic alcohol abuse accelerates brain aging and contributes to cognitive impairments, increasing the risk of brain changes [12,13,14,15,16].

Interestingly, several studies agree that alcohol misuse may raise the risk of dementia, though not specifically Alzheimer’s disease [17]. Key factors could be the potential reversibility of brain harm following abstinence from chronic alcohol intake, in contrast to the degenerative nature of Alzheimer’s disease, as well as the brain hallmark protein inclusions found in Alzheimer’s patients, which are not present in the brains of those with AUD. Alcohol also has a complex interplay with inflammation and immune activation, in particular cells damaged by the toxic effect of ethanol release cytokines, chemokines, and extracellular vesicles to recruit and activate macrophages and neutrophils [18,19]. Activated neutrophils further contribute to cell injury by producing reactive oxygen species (ROS), neutrophil extracellular traps (NETs), interleukin (IL) 8 and protease-promoting white blood cell infiltration. After phagocytosis of damaged cells, macrophages switch from a proinflammatory phenotype to a reparative phenotype by secreting fibrogenic cytokines, such as transforming growth factor beta (TGF-β) and platelet-derived growth factor B (PDGF-B), but also proinflammatory cytokines like IL-1 and tumor necrosis factor alpha (TNF-α).

Several studies suggest that moderate alcohol consumption can reduce the risk of certain autoimmune diseases (ADs) [20,21]. In contrast, heavy alcohol consumption is generally detrimental, exacerbating inflammation and increasing the risk of various health issues, including ADs. This dichotomy underscores the importance of moderation in alcohol consumption.

This narrative review aims to report updated evidence on the role of alcohol in ADs.

## 2. Autoimmune Diseases

ADs are a heterogeneous group of more than 100 pathological conditions affecting around 10% of the global population, with a higher frequency of affected women (13%) compared to men (7%) [22]. A combination of genetic predisposition and environmental exposures likely play a significant role in disease development [23,24]. In general, ADs occur when the body’s immune system mistakenly attacks its healthy cells, tissues, and organs, leading to a variety of symptoms and affecting multiple organs or tissues in the body. ADs are usually classified as organ-specific or non-organ-specific, depending on whether they affect one definite organ (e.g., thyroid disease) or several with systemic autoimmune activity (as in systemic lupus erythematosus) [25,26].

**Figure 1:**
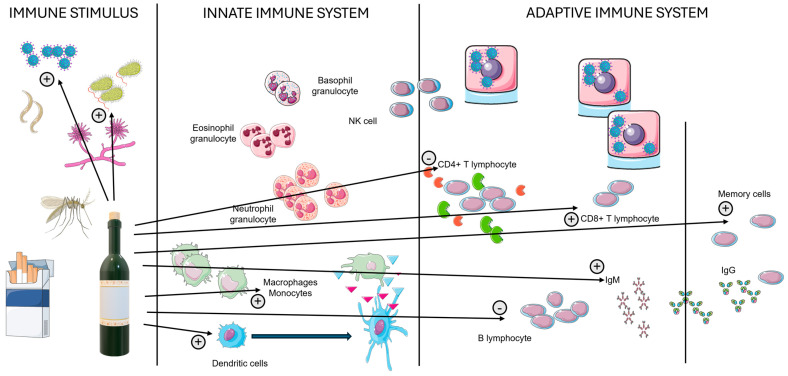
Immune system schematic process. Alcohol modulates immune responses in a dose-dependent way, showing differences when comparing heavy alcohol drinkers (black arrow) and moderate alcohol drinkers. Regarding innate immunity, heavy alcohol intake may increase inflammation, oxidative stress, and the risk of bacterial and viral infection, while moderate drinking supports the production of anti-inflammatory cytokines (IL-4, IL-10, TGF-β) and modulates the intestinal microbiota, reducing inflammation. Effects of alcohol consumption on adaptive immunity responses include impairment of T-cell and B-cell maturation, dysfunction (increased T-cell activation for cell death, increased B-cell production of IgM and IgA) and altered survival (reduced CD4 T cells, increased CD8 and T-memory cells). Further details on the effect of alcohol intake on immune responses are reported in Figure 2 and Table 1. Parts of the figure were drawn by using pictures from Servier Medical Art and Microsoft PowerPoint 365 Version 2112 (https://www.microsoft.com/microsoft-365). Servier Medical Art by Servier is licensed under a Creative Commons Attribution 3.0 Unported License (https://creativecommons.org/licenses/by/3.0/).

The immune system is a complex network of cells, tissues, and organs that work together to defend the body against harmful stimuli, such as bacteria, viruses, fungi, parasites, and cancerous cells [27]. The immune system can be broadly divided into two main components: the innate immune system (nonspecific) and the adaptive immune system (specific) [28]. The innate immune system provides the first line of defense and responds quickly to infections. It includes physical barriers (skin, mucous membranes, secretions), immune cells (phagocytes), a complement system (enhancing the ability of antibodies and phagocytic cells to clear pathogens), cytokines (promoting inflammation and recruiting immune cells to infection sites), and interferons (produced by virus-infected cells to protect neighboring cells from viral infection) [29] (see Figure 1).

The adaptive immune system provides a specific response to pathogens and has memory, allowing for a faster and more effective response upon subsequent exposure [30,31]. It involves B cells and T cells, which recognize specific antigens [32]. B cells produce antibodies (proteins that specifically bind to antigens, neutralizing pathogens or marking them for destruction by other immune cells), and when activated, they can differentiate into plasma cells (which produce and secrete large amounts of antibodies) or memory B cells (which provide long-term immunity). T cells include helper T cells (CD4+) that assist other immune cells (e.g., B cells and cytotoxic T cells) by releasing cytokines that enhance the immune response; cytotoxic T cells (CD8+), which directly kill infected or cancerous cells through the recognition of peptide antigens, provided in the form of major histocompatibility complex (MHC) I peptide complexes in mice and known as the human leukocyte antigen (HLA) in humans, by antigen-presenting cells (APCs); and regulatory T cells (Tregs), which suppress immune responses to maintain tolerance to self-antigens and prevent ADs [33]. When a pathogen invades, APCs like dendritic cells capture antigens and present them on their surface using MHC molecules. Helper T cells recognize antigens presented by APCs and become activated, secreting cytokines that stimulate other immune cells and help activate B cells, which then proliferate and differentiate into plasma cells and memory B cells [34,35]. Cytotoxic T cells kill infected cells, while antibodies produced by plasma cells neutralize pathogens or mark them for destruction by phagocytes and the complement system. Memory B and T cells are formed during the primary response and provide a faster, more robust response upon re-exposure to the same pathogen. Finally, Tregs and anti-inflammatory cytokines help downregulate immune responses to prevent excessive damage to the body’s tissues. After the pathogen is cleared, most immune cells undergo apoptosis, and the immune response is resolved to restore homeostasis. The innate and adaptive systems cooperate from the earlier phases, with complex mechanisms described in other interesting studies [36,37,38].

The immune system employs several mechanisms to regulate itself and avoid attacking the body’s tissues, thereby preventing ADs [39]. This regulation is critical to maintaining a balance between responding to harmful pathogens and tolerating the body’s cells [40,41]. Factors contributing to the breakdown of autoimmune tolerance include genetic predispositions, environmental triggers (such as infections or toxins), and dysregulation of immune checkpoints.

During their development in the thymus, T cells undergo a selection process whereby T cells that strongly recognize self-antigens are induced to programmed death through apoptosis (negative selection in the medulla), while those that moderately recognize self-antigens are retained (positive selection in the cortex) [42]. B cells undergo a similar process in the bone marrow: if a developing B cell strongly binds to self-antigens, it undergoes receptor editing, apoptosis, or anergy. Anergy is a state of reduced responsiveness that arises in developing B cells when they experience mild cross-linking of the B-cell receptor (BCR) [43].

In this state, anergic B cells have a shortened lifespan, characterized by diminished surface antibody receptor concentration and a less sensitive BCR, which hinders the activation of downstream signaling pathways. Although anergic B cells are unresponsive to direct BCR engagement, they can still be activated by other stimuli such as non-antigen-specific T-cell co-stimulation, lipopolysaccharide, or IL-4, suggesting that anergic B cells could potentially reactivate and have a role during inflammatory conditions. Tregs expressing the transcription factor Foxp3 suppress the activity of other immune cells that might attack self-antigens through direct cell-to-cell contact and by releasing inhibitory cytokines like IL-10 and TGF-β [44,45]. Peripheral immune cells that recognize self-antigens without the proper context (e.g., in the absence of infection or inflammation) can be induced to undergo apoptosis. Furthermore, there are “immune checkpoints” that prevent excessive immune responses (i.e., CTLA-4, LAG-3, and PD-1 inhibit T-cell activity and differentiation) [46].

Certain sites in the body, like the eyes, brain, and testes, are considered immune-privileged, as any immune response is tightly controlled to avoid damaging sensitive tissues such that in these organs, immune responses either do not proceed or proceed in a manner different from other areas [47]. Other mechanisms adopted to avoid the breakdown of autoimmune tolerance include the low expression of self-antigens in specific tissues, the necessity of appropriate co-stimulatory signals during antigen presentation to activate T cells and the role of a healthy microbiome. In particular, the microbiome maintains immune balance and prevents autoimmune reactions by modulating immune responses of Tregs, competing with harmful pathogens for nutrients and space, enhancing barrier function by strengthening the intestinal lining and preventing the leakage of antigens, and producing SCFAs and other metabolites useful for inflammation regulation [48,49,50].

The exact cause of ADs is not fully understood, but several factors are believed to contribute, including genetics, environment, hormones, and immune system dysregulation [51]. Common ADs include rheumatoid arthritis (RA) which affects joints, causing inflammation, pain, and potentially joint destruction; systemic lupus erythematosus (SLE), which affects multiple organs, including skin, joints, kidneys, and the nervous system; multiple sclerosis (MS), which targets mostly the central nervous system, leading to neurological symptoms such as muscle weakness, coordination issues, and vision problems; type 1 diabetes (DM1), which damages the beta cells that are the insulin-producing cells in the pancreas, resulting in high blood sugar concentration; Hashimoto’s thyroiditis (HT), which causes hypothyroidism by attacking the thyroid gland; Graves’s disease (GD), which leads to hyperthyroidism by stimulating the thyroid gland excessively; celiac disease (CD), which is triggered by the ingestion of gluten, leading to damage in the small intestine; psoriasis and other ADs affecting the skin and joints; and inflammatory bowel disease (IBD), which includes conditions like Crohn’s disease and ulcerative colitis, which cause chronic inflammation of the digestive tract [52,53].

Diagnosing ADs can be challenging due to the overlapping symptoms and typically involves a detailed medical history and physical exam followed by blood testing to detect autoantibodies, inflammatory markers, and other relevant indicators, as well as imaging tests such as X-rays, MRI, or CT scans, which assess the state of internal damage [54]. In some cases, biopsy tissue samples may be examined. Managing an autoimmune disease often requires a multidisciplinary approach, including regular monitoring and collaboration with healthcare providers. Treatments usually aim to manage symptoms and control the immune response with anti-inflammatory drugs and immunosuppressants. Sometimes, biological treatments that target specific parts of the immune system are available (e.g., adalimumab for RA or belimumab in severe SLE) [55]. It has been demonstrated that some patients benefit from diet and nutrition adjustments, regular exercise, and stress management techniques.

## 3. Alcohol Addiction and Autoimmune Diseases

### 3.1. Protective Role of Alcohol

Table 1 reports the main evidence on the protective role of alcohol in ADs. A recent article on the long-term health outcomes of moderate red wine consumption found that of a total of 74 studies evaluating a variety of health outcomes, there was no evidence of an association between red wine consumption and negative health outcomes, whereas 47 studies demonstrated an association between red wine consumption and positive health outcomes [20]. The concept of hormesis suggests that low doses of an otherwise harmful substance can have beneficial effects [21].

**Table 1:** Alcohol addiction and autoimmune disease literature evidence. Allergic rhinitis, AR; autoimmune thyroiditis, AITD; latent autoimmune diabetes in adults, LADA; multiple sclerosis, MS; primary biliary cholangitis, PBC; rheumatoid arthritis, RA; systemic lupus erythematosus, SLE; type 1 diabetes mellitus, T1DM; type 2 diabetes mellitus, T2DM.

Autoimmune Disease	Population	Results	Reference
**Autoimmune thyroiditis (AITD)**	5154 She ethnic minority people of Fujian province	Incidence of hypothyroidism and TPOAb positivity was decreased in case of alcohol consumption (defined as the average consumption of at least 35 g of alcohol per day).	[56]
	272 patients and 1088 controls	Moderate alcohol consumption is associated with reduction in the risk of hyperthyroidism irrespectively of age and gender.	[57]
	803 healthy women at risk of developing AITD	Alcohol consumption of >10 units/week may protect against the development of overt hypothyroidism.	[58]
	140 patients and 560 controls	Alcohol consumption protects against development of overt hypothyroidism irrespectively of sex and type of alcohol consumed.	[59]
	543 patients	Alcohol intake was not associated with risk of hyperthyroidism.	[60]
**Multiple sclerosis (MS)**	6619 patients and 7007 controls	Alcohol consumption exhibits a dose-dependent inverse association with MS.	[61]
	1717 patients (ages 15–19) with MS and 4685 healthy volunteers	Alcohol consumption in adolescence was associated with lower risk of developing MS in both sexes.	[62]
	10,249 patients, of which 215 had alcohol use disorders.	Alcohol use disorders in patients with MS results in significant increases in-hospital mortality and the length of the hospital stay and results in overexpenditure.	[63]
	923 patients	Higher total alcohol and red wine intake were associated with a lower cross-sectional level of neurologic disability in MS patients but increased T2LV accumulation.	[64]
	547 patients and 1057 healthy volunteers	After adjusting for measurement bias, confounding, and random error, alcohol consumption had a positive causal effect on the incidence of MS.	[65]
	210 patients	There is a significant association between consumption of hard liquor per day and risk of MS.	[66]
	2100 patients	No significant association between MS risk and alcohol consumption.	[67]
	258 patients		[68]
	146 patients and 294 controls	No significant association between primary progressive MS risk and alcohol consumption.	[69]
	Mouse models with experimental autoimmune encephalomyelitis	Alcohol significantly alters the course of MS differentially in males and females via effects on gut bacterial networks. This supports further need to evaluate dose and sex-specific alcohol effects in MS.	[70]
**Rheumatoid arthritis (RA)**	11,839 patients	Weekly alcohol consumption of <14 units per week does not appear to be associated with an increased risk of transaminitis.	[71]
	3353 patients and 2836 controls	The finding of a protective role of alcohol on risk of RA must be interpreted with caution from a clinical and public health perspective.	[72]
	1204 patients and 871 controls	Alcohol may protect against RA.	[73]
	873 patients and 1004 healthy controls	Alcohol consumption has an inverse and dose-related association with both risk and severity of RA.	[74]
	1238 patients	There is an association between alcohol consumption and both lower self-reported disease activity and higher health-related quality of life in female, but not in male RA patients.	[75]
	903 female patients	There is an association between long-term moderate alcohol drinking and reduced risk of RA in women.	[76]
	596 patients	There may be a deleterious effect of moderate consumption of alcohol on radiological progression in women, but not in men, with early RA.	[77]
	188 patients and 192 healthy volunteers	Confirmed the protective role of moderate alcohol consumption against RA, but alcohol was not associated with the severity of joint inflammation.	[78]
	197 patients	Moderate consumption of alcohol is associated with reduced risk of RA.	[79]
	174 patients	There is an association between alcohol consumption and markers of inflammation in RA patients prior to the occurrence of symptoms.	[80]
	158 female patients aged 55–69	Alcohol use did not influence the risk of RA.	[81]
	87 patients	Increased alcohol consumption is associated with an elevated risk of RA among women, but not in men.	[82]
	Alcohol-exposed mice	Alcohol-exposed mice have reduced Bcl6 and PD-1 expression as well as IL-21 production by TFH cells, preventing proper spatial organization of TFH cells to form TFH–B-cell conjugates in germinal centers, which ultimately impairs autoantibody formation and mitigates experimental autoimmune arthritis.	[83]
	Male DBA/1 mice	Low continued ethanol consumption delays the onset and halts the progression of collagen-induced arthritis by interaction with innate immune responsiveness.	[84]
**Systemic lupus erythematosus (SLE)**	1177 women	Alcohol may reduce SLE risk by decreasing circulating stem cell factor.	[85]
	282 female patients and 292 healthy volunteers	Alcohol intake is inversely associated with SLE risk.	[86]
	150 patients and 300 controls		[87]
	85 patients and 205 controls		[88]
	244 patients	There is an inverse association between moderate alcohol consumption (≥5 g or 0.5 drink/day) and SLE risk in women.	[89]
	127 patients	Confirmed decreased SLE risk with moderate alcohol consumption.	[90]
	114 patients and 228 controls	Alcohol consumption before SLE diagnosis is not associated with increased risk of SLE. Individuals who develop SLE are more likely to quit.	[91]
	125 patients and 125 controls	Alcohol was associated with neither increased risk nor a protective role.	[92]
	67 female patients		[93]
**Diabetes**	1841 T2DM and 140 T1DM	Moderate alcohol consumption reduces the risk of T1DM and T2DM.	[94]
	250 latent autoimmune diabetes in adults (LADA) and 764 T2DM	Alcohol intake may reduce the risk of type 2 diabetes and type 2-like LADA. but has no beneficial effects on diabetes-related autoimmunity.	[95]
**Allergic rhinitis (AR) and** **hypersensitivity reactions**	5870 women	Alcohol consumption is associated with an increased risk of developing perennial AR.	[96]
	3317 volunteers	Alcohol consumption was positively associated with aeroallergen sensitization.	[97]
	3460 adults	No association between alcohol consumption and nickel sensitization.	[98]
	734 subjects	Alcohol consumption leads to IgE-mediated immune responses rather than delayed-type hypersensitivity reactions such that it may prevent the development of contact sensitization.	[99]
**Primary biliary cholangitis (PBC)**	2576 patients and 2438 controls	Mild-to-moderate alcohol intake was negatively associated with PBC.	[100]
	103 patients and 100 controls		[101]
	200 patients and 200 controls	Alcohol appears to have an inverse relationship with PBC.	[102]

In the case of alcohol, low-to-moderate consumption (compared to sporadic, binge drinking, or heavy drinking patterns) may induce a mild stress response that strengthens the immune system’s ability to handle more significant stressors, potentially offering protection against ADs [103,104]. Evidence from both human and animal studies suggests that alcohol at low–moderate doses might have protective effects in ADs, highlighting a complex, dose-dependent relationship influenced by various factors such as duration and type of alcohol consumption, cultural background, and sex [105].

At low doses, the protective effects include altering the balance of anti-inflammatory innate and adaptive immune cells and modulating cytokine and chemokine concentrations, the composition of the gut microbiome, and the production of fatty acid metabolites, including SCFAs and polyunsaturated fatty acids (PUFAs) [106]. Moderate alcohol consumption has been shown to alter the production of cytokines, reducing proinflammatory cytokines like TNF-α, IL-8 and IL-6, while increasing anti-inflammatory cytokines like IL-10 [107,108]. This shift can lead to a reduced inflammatory state, potentially lowering the risk of autoimmune disease flare-ups. Alcohol can modulate the activity of immune cells such as T cells and macrophages, promoting a regulatory phenotype that is less likely to initiate autoimmune responses [109,110].

Different types of alcoholic beverages may have varying effects. For instance, red wine is particularly noted for its high content of polyphenols like resveratrol, which have antioxidant and anti-inflammatory properties contributing to the protective effects seen with moderate wine consumption [111,112,113,114]. Furthermore, the impact of alcohol on ADs can be influenced by cultural factors, including diet, lifestyle, and genetic predispositions. Mediterranean cultures, for example, where moderate wine consumption is common and paired with a healthy diet, may experience different outcomes compared to cultures with different drinking habits and dietary patterns [115,116]. As hormonal differences can influence immune responses and since ADs are more prevalent in women, studies have shown that moderate alcohol consumption may have more pronounced anti-inflammatory effects in women compared to men, potentially due to interactions with estrogen [117].

Population-based studies have found that moderate alcohol drinkers have a lower prevalence of ADs such as RA and SLE compared to both non-drinkers and heavy drinkers. Furthermore, affected patients presented with less severe symptoms and lower levels of systemic inflammation [85,118]. Old studies on the role of alcohol consumption in the development of contact sensitization found that it could be protective and suggested that this substance could lead to IgE-mediated immune responses rather than delayed-type hypersensitivity reactions [99]. Animal models of ADs, such as collagen-induced arthritis in mice, have shown that moderate alcohol consumption can reduce disease severity, decrease inflammatory cytokine concentrations, and alter immune cell function in a protective way [101]. Alcohol intake may be associated with a decreased risk of developing primary biliary cholangitis, a disease characterized by immune-mediated destruction of small and medium-sized intrahepatic bile ducts [102].

### 3.2. Alcohol Role as a Risk Factor for Autoimmune Diseases

Alcohol effects on the organism are complex and pleiotropic, with dose-dependent mechanisms influenced by various factors such as duration and type of alcohol consumption, cultural background, and sex. Its consumption can lead to chronic inflammation by promoting the production of proinflammatory cytokines and can alter the function of immune cells [119,120]. Moreover, alcohol causes dysbiosis, leading to an overgrowth of harmful bacteria and a decrease in beneficial bacteria, which may further trigger inflammation and affect immune system function [121].

Chronic consumption is associated with increased intestinal permeability, which allows toxins, undigested food particles, pathogens, and lipopolysaccharide (LPS) to pass through the gut barrier into the bloodstream. The production of acetaldehyde through oxidative metabolism is a primary contributor to alcohol-induced toxicity. Substantially, both alcohol and acetaldehyde can significantly trigger systemic inflammation through several mechanisms: (1) activating Toll-like receptors (TLRs) 2, 3, and 4, as well as the NOD-like receptor family pyrin domain-containing 3 (NLRP3) inflammasome complex in immune cells; (2) promoting bacterial overgrowth in the gastrointestinal (GI) tract, leading to increased production of LPS, a bacterial breakdown product; and (3) generating ROS and inducible nitric oxide synthase (iNOS), which compromise the integrity of gut tight junctions, resulting in the leakage of LPS into the bloodstream [122].

Notably, alcohol can activate the NLRP3 inflammasome, which plays a crucial role in the proinflammatory effects associated with chronic ethanol consumption [123]. This type of sterile inflammation may negate the beneficial effects of ethanol on the immune system and enhance its toxicity. The NLRP3 inflammasome is an important cytosolic complex of the innate immune system, primarily expressed in myeloid cells (monocytes and macrophages) and capable of triggering the production of IL-1β and IL-18 in response to danger and pathogen signals [124]. Activation of the NLRP3 inflammasome usually necessitates the assembly of NLRP3 with the apoptosis-associated speck-like protein containing a caspase recruitment domain (ASC, an adaptor protein) and procaspase 1 [124,125]. Chronic alcohol consumption upregulates and activates the NLRP3/ASC inflammasome, leading to caspase 1 activation and IL-1β release in the tissue, finally causing inflammation amplification [126].

On the other hand, it has been shown that ethanol interacts with the NLRP3 inflammasome by phosphorylating and thereby inhibiting the ASC adaptor protein, leading to an anti-inflammatory effect during acute ethanol exposure [127]. Purinergic signaling (notably through P2X7 receptors and the NLRP3 inflammasome) appears to be a decisive factor in the pathophysiology of alcoholic disease, as recent findings indicate that ethanol exposure can alter purinergic receptor concentration (including the upregulation of P2X7R in human macrophages), subsequently affecting interleukin production [128]. Hence, antagonists of purinergic receptors and the NLRP3 inflammasome might represent a new therapeutic option for treating alcoholic disease.

Alcohol consumption can also lead to epigenetic modifications such as DNA methylation and histone acetylation, altering the expression of genes involved in immune regulation and potentially triggering or exacerbating ADs [129]. Some epigenetic changes induced by alcohol can also be passed on to future generations, potentially affecting the immune system and disease susceptibility of offspring [130].

## 4. Autoimmune Thyroiditis

There are mainly two types of autoimmune thyroid disease (AITD): Hashimoto’s thyroiditis (HT) and Graves’s disease (GD) [131]. HT is a chronic autoimmune disorder characterized by lymphocytic infiltration and destruction of thyroid tissue, leading to hypothyroidism. It represents one of the most common ADs, with a higher incidence in women than men (10:1 ratio). Typical onset of the early symptoms is between 30 and 50 years of age [132]. Both genetic (HLA-DR3 and HLA-DR4 alleles, CTLA-4, PTPN22) and environmental (iodine intake, infections, stress, smoking, selenium deficiency and amiodarone, interferon) factors contribute to trigger immune dysregulation [133]. Immune mechanisms at the base of this pathology include the production of the autoantibodies anti-thyroid peroxidase (anti-TPO) and anti-thyroglobulin (anti-Tg), tissue infiltration by T cells (CD4+ and CD8+) and B cells, and the release of proinflammatory cytokines (e.g., IFN-γ, IL-1) promoting inflammation and apoptosis of thyroid cells, with progressive loss of thyroid follicular cells, fibrosis, and atrophy of the thyroid gland over time [134].

The most common symptoms of HT are fatigue, weight gain, cold intolerance, constipation, dry skin, hair loss, and bradycardia [135,136]. This disease is associated with an increased prevalence of other autoimmune conditions (e.g., type 1 diabetes, RA, pernicious anemia).

GD is an autoimmune disorder characterized by hyperthyroidism due to the production of thyroid-stimulating immunoglobulins (TSIs), which mimic thyroid-stimulating hormone (TSH) and bind to TSH receptors on the thyroid gland. Common symptoms and signs include weight loss, increased appetite, heat intolerance, sweating, irritability, tremors, thyroid, eye disease, goiter, and pretibial myxedema.

Approximately 25–50% of patients develop Graves’s ophthalmopathy, caused by inflammation and tissue remodeling around the eyes, leading to symptoms like bulging eyes (exophthalmos), double vision, eye pain, and redness. Genetic predisposition plays a significant role, with a higher prevalence in individuals with a family history of AITD, while environmental triggers, such as infections, stress, and smoking, may also contribute to disease onset.

Diagnosis of AITD is based on evaluation of symptoms and physical examination findings consistent with hypothyroidism or hyperthyroidism, altered serum TSH and free thyroxine (fT4) concentrations, presence of anti-TPO/anti-Tg antibodies/TSIs, and ultrasound findings. Fine-needle aspiration biopsy in atypical cases could be necessary to rule out malignancy [135,136]. Management of HT is mainly pharmacological with levothyroxine (synthetic fT4) guided by regular monitoring of TSH and fT4 concentrations [137]. Treatment of GD aims to control hyperthyroidism and its symptoms with antithyroid medications (e.g., methimazole, propylthiouracil), radioactive iodine-131 therapy to ablate thyroid tissue, and surgical thyroidectomy [138].

Management of GD ophthalmopathy may require corticosteroids, orbital decompression surgery, or other targeted therapies. It has been demonstrated that correction of dietary iodine deficiency, ending of smoking, and moderate alcohol intake play an important role in the management of autoimmune hypothyroidism [139].

Moderate alcohol consumption has been suggested to be beneficial in both types of AITD [56,58]. In particular, alcohol intake was found to protect against the development of autoimmune hypothyroidism, independently of sex or the type of alcoholic beverage (wine versus beer) [59]. Compared with the reference group with a mean consumption of 1–10 units of alcohol per week in the last year, it was found that not drinking at all seemed to be associated with a higher risk (OR 1.98), moderate drinking (11–20 units/week) with a lower risk (OR 0.41), and high consumption (≥21 units/week) showed no significant difference (0.90). The same author also reported that moderate ethanol drinking is associated with a dose-dependent reduction in the risk of developing GD, irrespective of age or gender [57]. In this case, the reference group drank 1–2 units of alcohol per week in the last year, while increasing dosages of ethanol caused better results, starting from the harmful abstinence (OR 1.73), up to ≥21 ethanol units/week (OR 0.22). These data on Graves’s disease are partially in contrast to previous data dated 2005 by Holm et al. involving young women [60]. Nonetheless, it should be noted that the older study was more focused on smoking and obesity as risk factors rather than alcohol consumption.

## 5. Multiple Sclerosis

Multiple sclerosis (MS) is a chronic, immune-mediated disorder of the central nervous system (CNS) characterized by demyelination, axonal injury, and neurodegeneration. The strongest genetic association is with the HLA-DRB1*15:01 allele, but other implicated genes include IL2RA, IL7R, and multiple loci identified through genome-wide association studies (GWAS) contributing to immune regulation [140]. Several environmental factors have been associated with MS, including Epstein–Barr virus (EBV) infection, low vitamin D concentrations, smoking, obesity, and living in temperate regions [141]. The immune mechanisms involve autoreactive CD4+ and CD8+ T cells that breach the blood–brain barrier (BBB), initiating inflammation, while B cells contribute through antigen presentation, cytokine production (e.g., IFN-γ, TNF-α), and antibody-mediated mechanisms [142,143].

Multiple sclerosis (MS) is primarily characterized by immune-mediated mechanisms, rather than by a specific set of autoantibodies commonly found in other ADs. However, research has identified several autoantibodies that may play a role in the pathogenesis of MS or serve as biomarkers for disease subtypes, including myelin oligodendrocyte glycoprotein (MOG) antibodies identified in a subset of patients, particularly those with optic neuritis, transverse myelitis, and some forms of demyelinating disease; antibodies against neurofascin proteins (NF155, NF186), found in more aggressive forms of MS and those exhibiting peripheral nerve involvement; potassium channel KIR4.1 antibodies; and anti-nuclear antibodies (ANAs), which are non-specific.

While some antibodies are helpful for differential diagnosis, such as anti-aquaporin 4 (AQP4), primarily associated with neuromyelitis optica spectrum disorders (NMOSDs), glial fibrillary acidic protein (GFAP) antibodies are typically found in patients with autoimmune GFAP astrocytopathy [144,145,146,147]. Oligoclonal bands (OCBs), which represent clonal expansion of B cells in the CNS, are considered a hallmark of MS and support the diagnosis, reflecting an ongoing immune response [148]. Histopathological characteristics are loss of myelin sheaths in white and gray matter, early axonal injury and transection, gliosis (proliferation of astrocytes) leading to scar formation in demyelinated areas, and lesions leading to MS plaques, typically found in periventricular white matter, optic nerves, the spinal cord, and brainstem [149,150].

Clinical presentation may follow different patterns: (1) relapsing–remitting MS (RRMS) is characterized by acute exacerbations (relapses) followed by periods of partial or complete recovery (remissions); (2) secondary progressive MS (SPMS) initially presents as RRMS, evolving afterwards to a phase of progressive neurological decline with fewer relapses; (3) primary progressive MS (PPMS) shows a steady progression of neurological symptoms from the onset, without distinct relapses and remissions; and progressive-relapsing MS (PRMS) is a progressive disease with superimposed relapses [151]. Diagnosis is based on the dissemination of lesions in time and space (MRI), supported by clinical history and neurological examination [152]. Cerebrospinal fluid (CSF) analysis may identify the presence of OCBs and an elevated immunoglobulin G (IgG) index [148]. The DMTs can reduce the frequency and severity of relapses, slowing disease progression and minimizing CNS damage [153]. This includes various treatments: interferon beta, glatiramer acetate, monoclonal antibodies (e.g., natalizumab, ocrelizumab), and oral agents (e.g., fingolimod, dimethyl fumarate). Rehabilitation and addressing symptoms such as spasticity, pain, fatigue, and bladder dysfunction may be useful to improve functional outcomes and quality of life.

It has been suggested that alcohol consumption may reduce the risk of MS and even exhibit a dose-dependent inverse association with MS [61,62]. Higher total alcohol intake has been associated with a lower cross-sectional level of neurologic disability in MS patients, but increased T2 hyperintense lesion volume (T2LV) [64]. On the other hand, a significant association between consumption of hard liquor per day and risk of MS was found (OR = 6.7, *p* = 0.026) [66]. Furthermore, MS patients with AUD have a very high in-hospital mortality rate (94.1%) and longer stays (2.4 days), generating overexpenditure (EUR 1116.9 per patient) [63]. Other studies found no significant association between MS risk and substance abuse and alcohol consumption [67,68,69]. Recent studies found that after adjusting for measurement bias, confounding, and random error, alcohol consumption has a positive causal effect on the incidence of MS [65].

In animal models, it has been demonstrated that alcohol ameliorates a murine model of MS (experimental autoimmune encephalomyelitis) in a sex-specific pattern through shifts in gut microbial networks [70]. Alcohol-fed males experienced significantly greater disease remission compared to alcohol-fed females and control-fed counterparts. An interesting recent study further discussed the epidemiology of alcohol consumption and MS [154].

## 6. Rheumatoid Arthritis

Rheumatoid arthritis (RA) is a chronic, systemic autoimmune disease primarily affecting synovial joints, leading to inflammation, pain, and progressive joint destruction [155]. It is characterized by synovial hyperplasia, increased synovial fluid, and the formation of pannus tissue. RA affects approximately 0.5–1% of the global population, with a higher prevalence in women than men (3:1 ratio). Onset typically occurs between the ages of 30 and 60, though it can present at any age. RA is initiated by an autoimmune response, wherein genetic (HLA-DRB1, PTPN22) and environmental (smoking, infections, and hormones) factors contribute to loss of tolerance to self-antigens [156,157]. Key elements include autoantibodies (rheumatoid factor and anti-citrullinated protein antibodies), involvement of T cells, B cells, macrophages, and synovial fibroblasts, which create a proinflammatory environment rich in cytokines such as TNF-α, IL-1, IL-6, and IL-17 and enzymes like matrix metalloproteinases (MMPs) able to degrade cartilage, while osteoclasts resorb bone [158].

Clinical features include symmetrical polyarthritis affecting small joints (hands, feet) and larger joints (knees, elbows), rheumatoid nodules, interstitial lung disease, cardiovascular involvement, fatigue, fever, and weight loss [159]. Usually, the diagnosis benefits from clinical evaluation (morning stiffness, joint swelling, symmetrical involvement), laboratory tests (inflammatory markers, autoantibodies) and imaging (ultrasound and MRI for early detection and X-rays for established disease) [160]. Pharmacological treatments include non-steroidal anti-inflammatory drugs (NSAIDs) and corticosteroids for symptom control and disease-modifying antirheumatic drugs (DMARDs), including methotrexate, sulfasalazine, and leflunomide. Sometimes, biological DMARDs are necessary: TNF inhibitors (e.g., etanercept, infliximab), IL-6 receptor antagonists (e.g., tocilizumab), and B-cell depleting agents (e.g., rituximab) [161].

Targeted synthetic DMARDs acting as JAK inhibitors (e.g., tofacitinib) are available. Furthermore, lifestyle modifications (smoking cessation, diet, and exercise), physical therapy, occupational therapy, and patient education are important in the management of this disease. Prognosis varies widely, as early and aggressive treatment improves outcomes. Complications include joint deformity, reduced function, and increased mortality, particularly due to cardiovascular disease.

Early studies on mice found that ethanol and its metabolite acetaldehyde may have protective properties against RA [84]. These results have been associated with the downregulation of leukocyte migration and decreased NF-kB activation. In an interesting study, 34,141 women born between 1914 and 1948 have been followed up from 1 January 2003 to 31 December 2009 [79]. A total of 197 patients presented with RA, and a 37% decrease in risk of RA was found among women who drank more than four glasses of alcohol (one glass = 15 g of ethanol) per week compared with women who drank < 15 g per week or who never drank alcohol (*p* = 0.04). Analysis of long-term alcohol consumption showed that women who reported drinking more than three glasses of alcohol per week halved the risk of RA compared with those who never drank. Furthermore, alcohol has been suggested to have an inverse and dose-related association with both risk and severity of RA [73,74], but this has been strongly debated, as no experiments have confirmed cause–effect relationships [162].

On the other hand, an older study that included 31,336 women aged 55–69 years, of which 158 cases presented RA, found that alcohol use did not influence the risk of this disease [81]. A recent article suggested that there is insufficient evidence for genetic causality between alcohol intake and arthritis [163]. Other experiments demonstrated an association between alcohol consumption and markers of inflammation, including C-reactive protein (CRP), fibrinogen, white blood cell (WBC) count, plasma viscosity, IL-6, and tumor necrosis factor receptor (TNFR), in RA patients before the occurrence of symptoms [80,164]. Interestingly, it has been shown that alcohol and its acetate alter the functional state of T follicular helper (T_FH_) cells in vitro and in animal models, thereby impairing autoantibody formation and leading to a reduction in experimental autoimmune arthritis [83]. By contrast, T cell-independent immune responses and passive models of arthritis were not affected by alcohol exposure.

Consistent with the protective role of alcohol in RA, various studies confirmed that low-to-moderate alcohol consumption in women prevents the onset of RA in a time-, dose-, and sex-dependent manner [76,118]. Alcohol consumption has been associated with lower self-reported disease activity and better health-related quality of life [75].

Compared with non-drinking, low and moderate alcohol consumption was dose-dependently associated with a reduced risk of anticitrullinated protein antibody (ACPA)-positive and ACPA-negative RA [72]. A three-way interaction has been observed between alcohol, smoking, and HLA-DRB1-SE concerning the risk of ACPA-positive RA.

On the contrary, a recent prospective study on a Chinese cohort found that increasing alcohol consumption was associated with an elevated risk of RA among women, but not in men [82]. These data have been supported by a European study that found that moderate alcohol consumption increased the radiological progression of RA in women, but not in men [77]. In general, the gender differences in the association between alcohol consumption and RA risk may be due to the effect of alcohol on estrogen signaling and the hypothalamic–pituitary–adrenal (HPA) axis.

Another interesting cross-sectional study found that alcohol is associated with lower concentrations of C-reactive protein, but not with less severe joint inflammation, suggesting that the pathophysiological mechanism underlying the effect of alcohol may consist of a systemic effect that might not involve the joints [78].

Despite the benefits of alcohol in AR, caution should be exercised, as many of the treatments used for rheumatic diseases can cause serious adverse hepatotoxicity if associated with ethanol. In fact, most guidelines recommend abstention from alcohol or suggest care with the use of certain disease-modifying therapies (e.g., methotrexate, NSAIDs, sulfasalazine, azathioprine). However, it should be noted that many studies suggest that low-to-moderate alcohol consumption (<14 units per week) does not appear to be associated with an increased risk of hepatic damage or transaminitis [71]. Baseline liver function tests should be taken into consideration in RA patients if alcohol abuse is suspected.

Interestingly, osteoarthritis patients who consume too much alcohol seem to have a higher risk of disease, fractures, and incident surgery because of its role in osteoblastic dysfunction resulting in diminished bone formation and reduced bone mineralization [165,166,167,168,169]. It has been suggested that alcohol causes bone problems through IL-6, which induces increased receptor activation of NFκB ligand, promoting granulocyte–macrophage colony-forming units and osteoclastogenesis [170,171]. Furthermore, it causes an imbalance in the catabolism of skeletal muscle proteins. The association between alcohol consumption and the development and exacerbation of osteoarthritis remains unclear, but the negative health impact of excessive alcohol consumption should be acknowledged when considered for management purposes [172].

## 7. Systemic Lupus Erythematosus

Systemic lupus erythematosus (SLE) is a chronic, multisystem autoimmune disease characterized by the production of autoantibodies and immune complex deposition, leading to widespread inflammation and tissue damage [173]. This pathology affects approximately 20–150 per 100,000 people globally, with higher prevalence in women (9:1 ratio) and typical onset between the ages of 15 and 45.

Genetic susceptibility (HLA-DR2, HLA-DR3, PTPN22, STAT4, IRF5), environmental triggers (ultraviolet light, Epstein–Barr virus, estrogen, procainamide, hydralazine), and immune dysregulation are at the base of its pathophysiology [174,175]. It is characterized by the production of anti-nuclear antibodies (ANAs), including anti-dsDNA, anti-Smith, anti-Ro, and anti-La antibodies, deposition of immune complexes in tissues, dysfunction in T cells, B cells, and dendritic cells, and impaired clearance of apoptotic cells [176]. Clinically, it may present as a variety of symptoms, including fatigue, fever, weight loss, cutaneous manifestations like malar rash (“butterfly” rash), discoid lesions, photosensitivity, oral and nasal ulcers, arthralgia, arthritis, myalgia, nephritis (presenting as proteinuria, hematuria, and renal impairment), pericarditis, myocarditis, Libman–Sacks endocarditis, pleuritis, interstitial lung disease, pulmonary hypertension, seizures, psychosis, cognitive dysfunction, peripheral neuropathy, anemia, leukopenia, thrombocytopenia, and antiphospholipid syndrome [177].

Diagnosis is based on a combination of clinical and immunological features, such as the 2019 EULAR–ACR classification criteria, positiveness to autoantibodies, compatible laboratory tests (low complement C3/C4 concentrations during active disease, elevated CRP, proteinuria, hematuria, cytopenia), and imaging and biopsy tests to assess organ involvement (echocardiograms, renal ultrasound, renal biopsy) [178,179,180]. Management is complex, and the disease progression varies from mild to severe, with improved outcomes with early diagnosis and advances in treatment, although morbidity remains significant [181,182]. Pharmacological treatment includes NSAIDs for mild symptoms, hydroxychloroquine (an antimalarial drug used for skin and joint symptoms), corticosteroids for moderate-to-severe disease flares, immunosuppressants (methotrexate, azathioprine, mycophenolate mofetil, cyclophosphamide) in cases of severe or organ-threatening disease, biological drugs (anti-BLyS belimumab for refractory cases, anti-CD20 rituximab off-label), and anticoagulants for those patients with antiphospholipid syndrome. Patients also benefit from avoidance of UV exposure using sunscreens and regular monitoring of disease activity and organ function.

It was reported decades ago that alcohol has beneficial effects in patients affected by SLE [86,87,92]. However, lately, some studies found no benefits or even increased risk of SLE in cases of excessive alcohol consumption [91,93,183]. These studies were mostly case–control or small-sample experiments with evident limitations. The early data have been confirmed by more recent studies that also found an inverse association between moderate alcohol consumption and SLE risk in women [88,89,90,184,185]. Increased daily alcohol consumption has been associated with a decrease in urinary neopterin, a marker of macrophage activation and SLE disease activity [186]. The mechanisms at the base of alcohol intake’s inverse relationship to the risk of SLE are under study, and it has been suggested that a major role is played by its ability to reduce cellular responses to immunogens and suppress the synthesis of proinflammatory cytokines TNF, IL-6, and IL-8 [109]. Other authors suggested that a plausible mechanism relies on a decrease in circulating stem cell factor [85]. Furthermore, antioxidants like resveratrol in wine and humulones in beer and alcohol-induced epigenetic changes can also potentially impact immune homeostasis [129,187,188,189]. An interesting study conducted in Japan identified an interaction between alcohol consumption and the N-acetyltransferase 2 (NAT2) genotype, as well as between NAT2 and alcohol consumption, indicating that individuals with the NAT2 rapid acetylation genotype and/or higher alcohol consumption have a lower risk of SLE compared to NAT2 slow acetylation [190]. This research highlighted the importance of incorporating genetic and metabolic information in studies on the management of SLE, suggesting that alcohol intake and genetic variations in liver-metabolizing enzymes may impact individual vulnerability to SLE.

Despite the abundant evidence, most of the papers published in recent years agree that alcohol consumption’s effect on SLE is still controversial and needs more research [191]. Furthermore, alcohol’s role in SLE multiorgan involvement should be considered. Indeed, it has been demonstrated that alcohol consumption is significantly associated with the presence of cutaneous damage [192].

## 8. Diabetes

Autoimmune diabetes, also known as type 1 diabetes mellitus (T1DM), is a chronic condition characterized by immune-mediated destruction of insulin-producing beta cells in the pancreas [193,194]. Autoimmune diabetes is a complex interplay of genetic (HLA-DR3-DQ2, HLA-DR4-DQ8, INS, PTPN22, and CTLA4), environmental (smoke, alcohol abuse, vitamin D deficiency, enterovirus infection, exposure to cow’s milk proteins or gluten), and immunological factors leading to the selective destruction of pancreatic beta cells and resulting in absolute insulin deficiency [195,196,197]. The main autoantibodies found in this autoimmune disease are islet cell autoantibodies (ICA), glutamic acid decarboxylase autoantibodies (GADA), GAD65, insulin autoantibodies (IAAs, more common in young children), tyrosine phosphatase-like insulinoma antigen 2 autoantibodies (IA-2As), and zinc transporter 8 autoantibodies (ZnT8As) [198,199].

These antibodies are fundamental for the diagnosis of this disease [200]. Classical symptoms include polyuria, polydipsia, polyphagia, and unexplained weight loss, but diabetic ketoacidosis is a common initial presentation in children and adolescents. Sometimes, it can manifest itself as a slower-progressing form of autoimmune diabetes in adults, often initially misdiagnosed as type 2 diabetes, known as latent autoimmune diabetes in adults (LADA) [201]. Characteristic histopathological findings are infiltration of pancreatic islets by immune cells and progressive loss of beta cells. The management of T1DM comprises lifelong exogenous insulin, regular blood glucose monitoring, and HbA1c testing to assess glycemic control [202]. Several gene-level interventions and immunomodulatory therapies are being investigated to restore or preserve residual beta-cell function and prevent progression [203].

Moderate alcohol consumption is associated with a reduced risk of type 2 diabetes and T1DM in adults. while high alcohol consumption carries an increased risk of harmful effects. but no higher risk of diabetes [94,204]. On the other hand, ethanol’s protective effect could be limited to men. Interestingly, the protective role has also been confirmed in LADA patients with low GADA concentrations (every 5 g of alcohol consumed per day reduced the risk of LADA by 6%), while no association was found in those patients with high GADA concentrations [95]. These findings may reflect the beneficial effects of ethanol in ameliorating insulin sensitivity and reducing inflammation [108,205,206]. Data suggest that LADA risk could be reduced by 60% by implementing healthy lifestyle changes including normal weight, non-smoking, physical activity, moderate alcohol consumption, and a healthy diet [207].

## 9. Allergic Rhinitis

Allergic rhinitis (AR) is an IgE-mediated inflammatory condition of the nasal mucosa triggered by exposure to allergens [208]. AR affects 10–30% of the global population, with higher rates in developed countries. Its onset is usually in childhood or adolescence, but can occur at any age. Family history of atopy, urban living, and exposure to indoor allergens (e.g., dust mites, and pet dander) are recognized as risk factors [209]. It manifests as sneezing, nasal congestion, rhinorrhea, and itching.

Associated conditions may be conjunctivitis, sinusitis, asthma, and atopic dermatitis. Its pathophysiology includes various stages: (1) during the sensitization phase, an initial exposure to allergens leads to the production of specific IgE antibodies by B cells, which bind to high-affinity receptors (FcεRI) on mast cells and basophils; (2) in the early phase upon re-exposure, allergens cross-link IgE on mast cells, triggering degranulation with release of histamine, leukotrienes, and prostaglandins causing immediate symptoms (sneezing, itching, rhinorrhea); and (3) during the late phase, 4–8 h post-exposure, recruitment of eosinophils, T cells, and other inflammatory cells to the nasal mucosa results in nasal congestion and hyperreactivity. Skin-prick tests or specific IgE blood tests are useful to identify causative allergens [210].

Environmental control measures to reduce exposure to identified allergens are necessary, but patients may benefit from antihistamines (e.g., cetirizine, loratadine), intranasal corticosteroids (e.g., fluticasone, mometasone), decongestants, leukotriene receptor antagonists (e.g., montelukast), and sometimes intranasal anticholinergics (e.g., ipratropium, in case of rhinorrhea) [211,212]. Subcutaneous immunotherapy (SCIT) for gradual desensitization through regular allergen injections or sublingual immunotherapy (SLIT), which uses allergen tablets or drops administered under the tongue, may be used as a potential cure for this chronic disease [213].

An interesting Danish population-based cohort study evaluated the role of alcohol consumption in 5870 young women (aged 20–29 years) [96]. A total of 831 (14%) women developed seasonal AR and 523 (9%) women developed perennial AR. Only the perennial disease was positively associated with alcohol consumption and only in women drinking more than 14 drinks/week compared with women drinking, i.e., less than one drink/week (OR 1.78). Ethanol intake (both alcohol abuse and moderate alcohol consumption) is associated with increased total serum IgE concentrations and aeroallergen sensitization, but it seems not to be associated with or sometimes protective for the development of contact sensitization [97,98,99,214]. On the other hand, more recent studies did not observe a causal relationship between alcohol consumption or volume of ethanol intake and the prevalence of asthma or allergic diseases, so there may be specific patterns to be found regulating the immune response in these pathologies [215].

## 10. Primary Biliary Cholangitis

Primary biliary cholangitis (PBC), formerly known as primary biliary cirrhosis, is a chronic autoimmune liver disease characterized by the progressive destruction of the small bile ducts within the liver, leading to cholestasis, liver fibrosis, cirrhosis, and liver failure if left untreated [100,216]. The exact cause of PBC is not fully understood, but it is believed to involve a combination of genetic predisposition, epigenetic changes, immune dysregulation and environmental triggers (urinary tract infections, exposure to chemicals, and certain medications) [217]. An immune-mediated assault targets the epithelial cells lining the small bile ducts, leading to chronic nonsuppurative destructive cholangitis, which results in the damage of bile ducts, cholestasis, and accumulation of toxic bile acids within the liver [218].

Persistent cholestasis and bile duct destruction promote inflammation and fibrosis in the liver parenchyma, which over time can lead to the development of cirrhosis. Early symptoms include fatigue, itching, xanthomas, and xanthelasmas [219]. Over time, the clinical manifestations include jaundice, hepatomegaly and splenomegaly, portal hypertension, variceal bleeding, and ascites. Diagnosis benefits from autoantibodies tests looking for antimitochondrial antibodies (AMAs; M2 are highly specific, detectable in less than 1% of healthy subjects); present in 90% of patients, antinuclear antibodies (ANAs), present in 30–50% of PBC patients, usually with patterns of nuclear dots (Sp100), or nuclear rim (gp210), as well as other less common antibodies like anti-small nuclear ribonucleoproteins (anti-SnRNPs) and anti-centromere antibodies (ACAs) [220,221,222,223]. Liver function tests (alkaline phosphatase and gamma-glutamyl transferase, bilirubin and transaminases) are useful. Liver biopsy shows chronic nonsuppurative destructive cholangitis and granulomatous inflammation centered on bile ducts, while ultrasound, MRCP (magnetic resonance cholangiopancreatography), and other imaging modalities help exclude other causes of cholestasis and assess liver structure [224].

The management of this disease is complex, as it involves the use of ursodeoxycholic acid (UDCA) to improve bile flow and delay disease progression, obeticholic acid (in patients who do not respond adequately to UDCA), antipruritic agents (cholestyramine, rifampicin, or naltrexone), fat-soluble vitamin supplementation to prevent deficiencies due to malabsorption, management of complications, and in extreme cases liver transplantation [219,225]. Early diagnosis and treatment with UDCA have significantly improved the prognosis of PBC. With appropriate management, many patients can live for decades without progressing to liver failure; however, for those who do progress to cirrhosis, liver transplantation remains a viable option with excellent outcomes.

One of the first studies found no significant difference in alcohol intake between 1032 cases and 1041 controls based on a rudimentary measure of ≥12 standard drinks per week over a lifetime [226]. On the other hand, more recent studies suggest that mild-to-moderate alcohol intake may be associated with a decreased risk of developing PBC [101,227].

In 2022, French and colleagues published the first study thoroughly evaluating the intake of alcohol in PBC cases from adolescence until the age of PBC diagnosis [102]. They found an inverse association between alcohol and PBC development, with cases reporting significantly less alcohol intake before onset, with dose–response trends. While the mechanism at the base of this association remains unclear, there are many proposals like the possible loss of natural killer cell activity, changes in immunoglobulin concentrations and alterations in T helper 1 (Th1)- and Th2-mediated immunity [102,110,228,229]. While some studies found negative associations between PBC and moderate alcohol intake [101,227], more recent studies found no direct effects and no genetic causal effects of ethanol on PBC [230].

Evidence suggests that alcohol consumption may also be protective because of its role in reducing the risk of autoimmune hepatitis [230,231].

It has been highlighted that since patients with recognized liver disease may underreport alcohol consumption prior to disease onset, most of these studies may suffer from recall bias due to the stigma concerning the intake of alcohol.

## 11. Discussion

While moderate alcohol consumption can be part of a healthy lifestyle, excessive consumption can lead to adverse health effects, emphasizing the importance of balance. Alcohol and in particular wine play a notable role in the Mediterranean diet, contributing both to the cultural and nutritional aspects of this eating pattern [232,233]. The Mediterranean diet emphasizes moderate consumption of wine, which is generally defined as one to two glasses (15–30 g of ethanol) per day for men and one glass per day for women [234]. Moderate wine consumption, particularly of red wine, has been associated with health benefits partially associated with the antioxidant content such as resveratrol and flavonoids, but also to various mechanisms, including immuno-inflammatory modulation and epigenetic changes [112].

Current evidence indicates a dose-dependent relationship between alcohol consumption and the severity of various ADs, including AITD, MS, RA, SLE, T1DM, LADA, AR and PBC. Low-to-moderate alcohol intake appears to exert protective effects, whereas higher levels of consumption can lead to addiction and exacerbate symptoms, worsening the outcomes of ADs [122]. The precise mechanisms by which low-to-moderate alcohol intake alleviates AD symptoms are not yet fully understood. Emerging studies suggest that ethanol may have a systemic immunomodulatory effect, potentially altering the balance of anti-inflammatory innate and adaptive immune cells, as well as cytokines and chemokines. Although the specific mechanisms have yet to be further characterized, the modulation of cytokine production via the NF-κB or NLRP3 pathways appears to be at the base of ethanol’s immunomodulatory effects [104].

Additionally, alcohol might influence the composition of the gut microbiome and the production of fatty acid metabolites, such as SCFAs and polyunsaturated fatty acids (PUFAs) [235]. At high doses, alcohol disrupts the gut barrier, leading to dysbiosis and an increase in bacterial wall products like LPS [124,126]. This LPS can activate TLRs on immune cells, resulting in elevated proportions of monocytes, T cells, cytokines, and immunoglobulins, alongside a reduction in B cells contributing to organ damage. Conversely, low-to-moderate alcohol consumption has been shown to reduce the risk and progression of ADs [104]. Although the exact mechanism remains unclear, it is suggested that low-to-moderate alcohol intake may reduce inflammation by increasing amounts of beneficial gut microbes, as well as elevated concentrations of acetate, PUFAs, high-density lipoprotein (HDL), and nitric oxide (NO). Furthermore, the gut microbiome could be manipulated to improve therapy and to derive greater benefit from existing therapies in ADs [236].

To calculate the amount of a substance in food or drinking water that can be consumed daily over a lifetime without presenting an appreciable health risk, the “no observed adverse effect level” is usually divided by a safety factor of 100 to address the uncertainties associated with the available scientific data [237]. The ADI for ethanol seems to be 2.6 g/day, deduced from morbidity and mortality rates due to liver fibrosis, but establishing a specific ADI of alcohol is inherently complex due to the multitude of biological mechanisms and pathways influenced by it [5,238].

Ethanol affects nearly every organ system in the body, and its impact varies depending on factors such as dosage, duration of exposure, genetic predisposition, and individual health status [1,7]. These factors contribute to the challenge of establishing a universal guideline that balances the potential benefits with the known risks. One of the primary reasons why a definite ADI for ethanol remains elusive is the dual nature of alcohol’s effects on human health. While moderate alcohol consumption has been associated with certain protective effects such as reduced risk of coronary artery disease and improvements in HDL cholesterol concentrations, these benefits must be carefully weighed against the numerous risks [239].

Alcohol consumption, even at low-to-moderate quantities, has been linked to an increased risk of various cancers, liver disease, and neurodegenerative conditions [240,241]. Moreover, the potential for addiction and the social consequences of alcohol misuse further complicate the establishment of a safe threshold [9,242]. Factors such as age, sex, genetic makeup, existing health conditions, and even lifestyle choices like diet and exercise can influence how an individual metabolizes and responds to alcohol. For instance, certain genetic polymorphisms in alcohol dehydrogenase (ADH) and aldehyde dehydrogenase (ALDH) enzymes can affect the rate at which alcohol is metabolized, leading to varying intensities of exposure to acetaldehyde [243,244]. These variations can result in different levels of risk of alcohol-related harm across populations, making it difficult to recommend a one-size-fits-all ADI. In light of these complexities, it is essential to adopt a cautious approach when considering alcohol consumption, even in the context of its potential benefits [245]. Furthermore, when it comes to alcohol abuse, it causes important risks to health, so it should be noted that the therapeutic effects at low dosages are difficult to apply in clinical contests, where alcohol dependence may be a direct and difficult-to-manage possible consequence of an intake recommendation [246]. Interestingly, as some therapeutic approaches to alcohol dependence are being tested, reductions in WHO risky drinking levels during treatment seem to reflect meaningful reductions in alcohol-related consequences and improved functioning [247]. The findings that suggest alcohol may have a protective role in certain diseases are intriguing, but they must be interpreted with caution.

## 12. Conclusions

In conclusion, despite the promising role of alcohol in preventing and reliving ADs, the risks associated with alcohol consumption, including the potential for abuse, addiction, and long-term health consequences, should always be at the forefront of any recommendations. Future research should focus on identifying and modulating the specific pathways and mechanisms that confer protective effects of alcohol without the need for ethanol consumption. For example, exploring pharmacological or dietary interventions that mimic the beneficial effects of moderate alcohol intake on cardiovascular health or ADs without exposing individuals to the risks associated with ethanol could be a promising avenue.

Additionally, research should aim to better understand the genetic and environmental factors that influence individual responses to alcohol to develop personalized management guidelines for alcohol consumption. Ultimately, the goal should be to harness the positive aspects of alcohol-related findings while minimizing the risks. This could also involve identifying alternative compounds or strategies that activate the same protective pathways as alcohol, but without its detrimental effects. Until such strategies are developed and validated, it is crucial to approach alcohol consumption with caution and to prioritize public health over the potential benefits of moderate drinking.

## Figures and Tables

**Figure 2:**
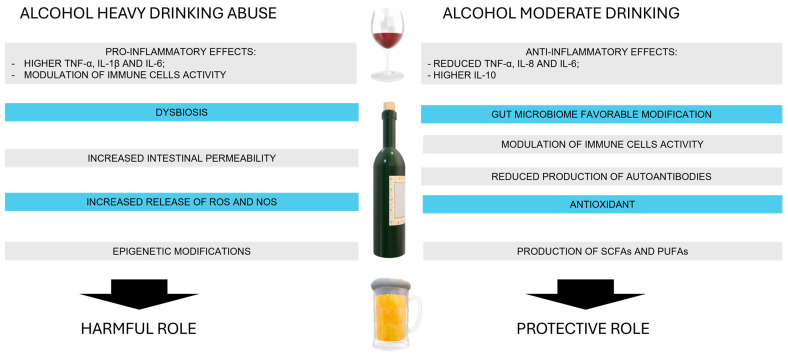
Harmful and protective role of alcohol in immune diseases. Parts of the figure were drawn by using pictures from Servier Medical Art and Microsoft PowerPoint 365 Version 2112 (https://www.microsoft.com/microsoft-365).

## Data Availability

Not applicable, since this is a review study.
